# Anti-Restenotic Roles of Dihydroaustrasulfone Alcohol Involved in Inhibiting PDGF-BB-Stimulated Proliferation and Migration of Vascular Smooth Muscle Cells

**DOI:** 10.3390/md13053046

**Published:** 2015-05-15

**Authors:** Pei-Chuan Li, Ming-Jyh Sheu, Wei-Fen Ma, Chun-Hsu Pan, Jyh-Horng Sheu, Chieh-Hsi Wu

**Affiliations:** 1 Department of Pharmacology and Pharmaceutical Sciences, School of Pharmacy, University of Southern California, Los Angeles, CA 90089, USA; E-Mail: lipeichu@usc.edu or u9955852@cmu.edu.tw; 2 School of Pharmacy, China Medical University, Taichung 404, Taiwan; E-Mail: soybean13mtdtw@gmail.com; 3 School of Nursing, China Medical University, Taichung 404, Taiwan; E-Mail: lhdaisy@mail.cmu.edu.tw; 4 Department of Pharmacy, Taipei Medical University, Taipei 110, Taiwan; E-Mail: panch@tmu.edu.tw; 5 Department of Marine Biotechnology and Resources, National Sun Yat-sen University, Kaohsiung 804, Taiwan; E-Mail: sheu@mail.nsysu.edu.tw

**Keywords:** dihydroaustrasulfone alcohol, anti-restenosis, neointimal hyperplasia, marine origin

## Abstract

Dihydroaustrasulfone alcohol (DA), an active compound firstly isolated from marine corals, has been reported to reveal anti-cancer and anti-inflammation activities. These reported activities of DA raised a possible application in anti-restenosis. Abnormal proliferation and migration of vascular smooth muscle cells (VSMCs) and the stimulation of platelet-derived growth factor (PDGF)-BB play major pathological processes involved in the development of restenosis. Experimental results showed that DA markedly reduced balloon injury-induced neointima formation in the rat carotid artery model and significantly inhibited PDGF-BB-stimulated proliferation and migration of VSMCs. Our data further demonstrated that translational and active levels of several critical signaling cascades involved in VSMC proliferation, such as extracellular signal-regulated kinase/mitogen-activated protein kinases (ERK/MAPK), phosphatidylinositol 3-kinase (PI3K)/AKT, and signal transducer and activator of transcription (STAT), were obviously inhibited. In addition, DA also decreased the activation and expression levels of gelatinases (matrix metalloproteinase (MMP)-2 and MMP-9) involved in cell migration. In conclusion, our findings indicate that DA can reduce balloon injury-neointimal hyperplasia, the effect of which may be modulated through suppression of VSMC proliferation and migration. These results suggest that DA has potential application as an anti-restenotic agent for the prevention of restenosis.

## 1. Introduction

Balloon angioplasty-induced restenosis is characterized by platelet aggregation, the release of growth factors, inflammation, abnormal proliferation and migration of vascular smooth muscle cells (VSMCs) within the media layer of arterial wall, and extracellular matrix (ECM) remodelling. Among these events, VSMC proliferation and migration have been believed to play a critical role involved in the development of atherosclerosis and restenosis [1,2]. Platelet-derived growth factor (PDGF) is one of the major regulators involved in promoting the proliferation and migration of VSMCs [3,4,5]. Among the different isoforms of PDGF, PDGF-BB triggers the strongest activation in the downstream signaling of the PDGF receptor [2], and most of its biological effects are initiated by several intracellular signaling pathways, such as extracellular signal-regulated kinase (ERK)-mitogen-activated protein kinases (MAPK), phosphatidylinositol 3-kinase (PI3K)/AKT, and signal transducer and activator of transcription (STAT) [6,7], to contribute VSMCs proliferation and migration.

**Figure 1:**
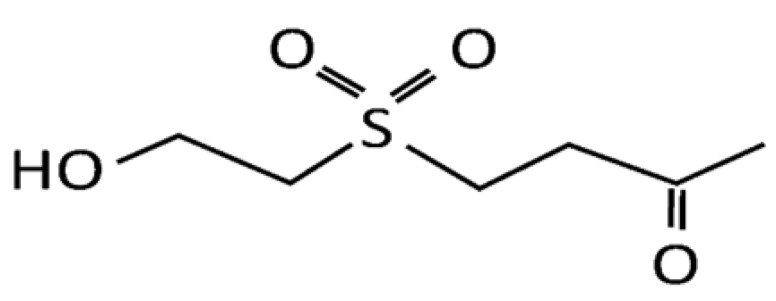
The chemical structure of dihydroaustrasulfone alcohol (DA).

Recent studies indicated a novel application of marine origins in preventing biological activities, such as anti-cancer [8], anti-inflammation [9], and anti-restenosis [10]. For example, soft coral-derived natural marine compounds, such as Capnellene and lemnalol, have been shown to attenuate the chronic constriction injury-induced neuropathic pain and inflammatory/analgesic effects [11,12]. Dihydroaustrasulfone alcohol (DA; Figure 1) isolated from soft corals has been shown to exhibit the neuro-protective [13], anti-inflammatory [14], and anti-cancer activities [8]. Wen *et al.* [9] showed that DA not only exhibited anti-inflammatory activity *in vitro* but also contained potent therapeutic effect for treating neuropathic pain and multiple sclerosis in rats. In the present study, we attempted to investigate whether DA possessed inhibitory effects and activity to prevent PDGF-BB-stimulated VSMCs proliferation and migration as well as balloon injury-induced neointimal hyperplasia.

## 2. Results and Discussion

### 2.1. Dihydroaustrasulfone Alcohol Inhibited Platelet-Derived Growth Factor (PDGF)-BB-Induced Vascular Smooth Muscle Cell (VSMC) Proliferation

**Figure 2:**
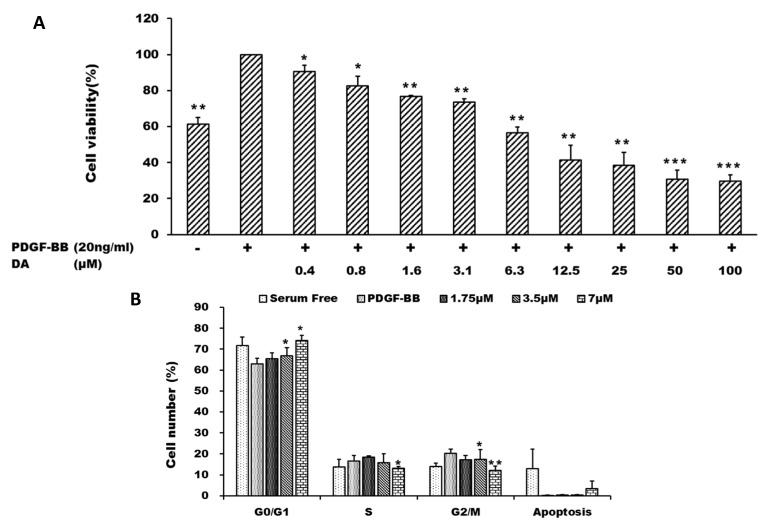
Dihydroaustrasulfone alcohol inhibits platelet-derived growth factor (PDGF)-BB-induced vascular smooth muscle cell (VSMC) proliferation. VSMCs were stimulated with PDGF-BB in the presence of different doses of DA for 24 h. (**A**) Cell viability of VSMCs was measured by using MTT assay; (**B**) The changes of cell cycle distribution were analyzed by flow cytometry. * *p* < 0.05; ** *p* < 0.01 and *** *p* < 0.001 compared with the control group (PDGF-BB alone).

DA has been demonstrated to induce cell cycle arrest and growth suppression in cancer cells [8]. VSMCs isolated from rat thoracic aorta were used to evaluate the effects of DA in the present study. We examined the effects of DA on PDGF-BB-induced proliferation of VSMCs by using MTT assay. VSMCs were stimulated by PDGF-BB (20 ng/mL) in the presence of different concentrations (0.4–100 μM) of DA for 24 h. DA inhibited PDGF-BB-induced VSMC proliferation in a concentration-dependent manner, and the IC_50_ value of DA was about 7 μM (Figure 2A). Subsequently, we determined the effect of DA on cell cycle progression by flow cytometry. VSMCs were stimulated by PDGF-BB and co-treated with different concentrations of DA (1.75, 3.5 and 7 μM). The results showed that DA induced cell cycle arrest at G0/G1 phase, and no significant sub-G1 population was observed (Figure 2B). Furthermore, analysis of annexin V-PI double staining study also supported that DA treatment did not cause evident apoptotic effect under the experimental condition (data not shown). These results indicate that the anti-proliferation effect of DA is likely due to its cytostatic effect.

### 2.2. Dihydroaustrasulfone Alcohol Inhibits PDGF-BB-Induced VSMC Migration

**Figure 3:**
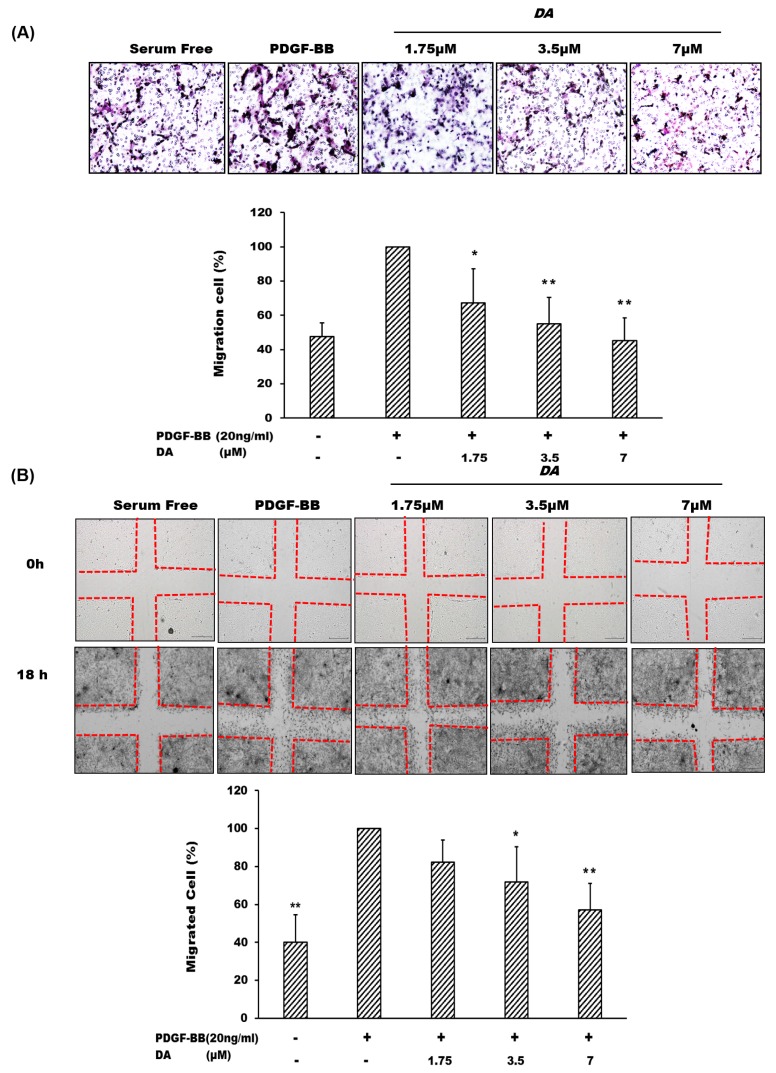
Dihydroaustrasulfone alcohol inhibited PDGF-BB-mediated cell migration of VSMCs. (**A**) Transwell and (**B**) wound healing assays were applied to test the potential regulation of DA on cell migration. VSMC migration was stimulated by 20 ng/mL of PDGF-BB in the presence of various doses (1.75, 3.5 and 7 μM) of DA for 18 h. The images were taken at 200× magnification. Results were expressed as the number of migrated cells relative to the control groups. * *p* < 0.05 and ** *p* < 0.01 compared with the control group (PDGF-BB alone).

Cell migration is defined as the movement of individual cells from one location to another. Two methods, the transwell and the wound healing assays, were introduced to examine the inhibitory effect of DA on VSMC migration. Our data indicated that PDGF-BB can markedly induce VSMCs migration as compared with that of the group treated with serum-free alone, whose effect can be markedly attenuated by DA co-treatment (Figure 3A). Similarly, experimental results obtained from the wound healing assay shows that DA also markedly suppressed VSMC migration stimulated by PDGF-BB treatment (Figure 3B).

### 2.3. Effects of Dihydroaustrasulfone Alcohol on the Proliferative and Migration-Associated Proteins in VSMCs

**Figure 4:**
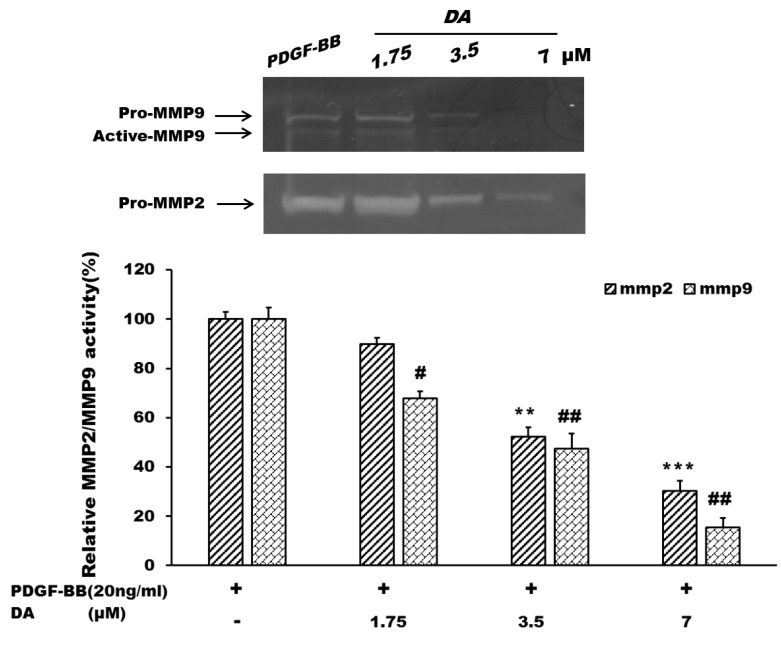
The regulations of Dihydroaustrasulfone alcohol on gelatinase activation. VSMCs were seeded on six-well plates (3 × 10^5^ cells/well) and stimulated with PDGF-BB (20 ng/mL) for 24 h. The cells were then treated with various concentrations of DA (1.75, 3.5 and 7 μM). The conditioned medium was collected to examine the activities of MMP-2 and MMP-9 by gelatin zymography. ** *p* < 0.01 and *** *p* < 0.001 compared with the control group (PDGF-BB alone). **^#^** *p* < 0.05 and **^##^** *p* < 0.01 compared with the control group (PDGF-BB alone).

**Figure 5:**
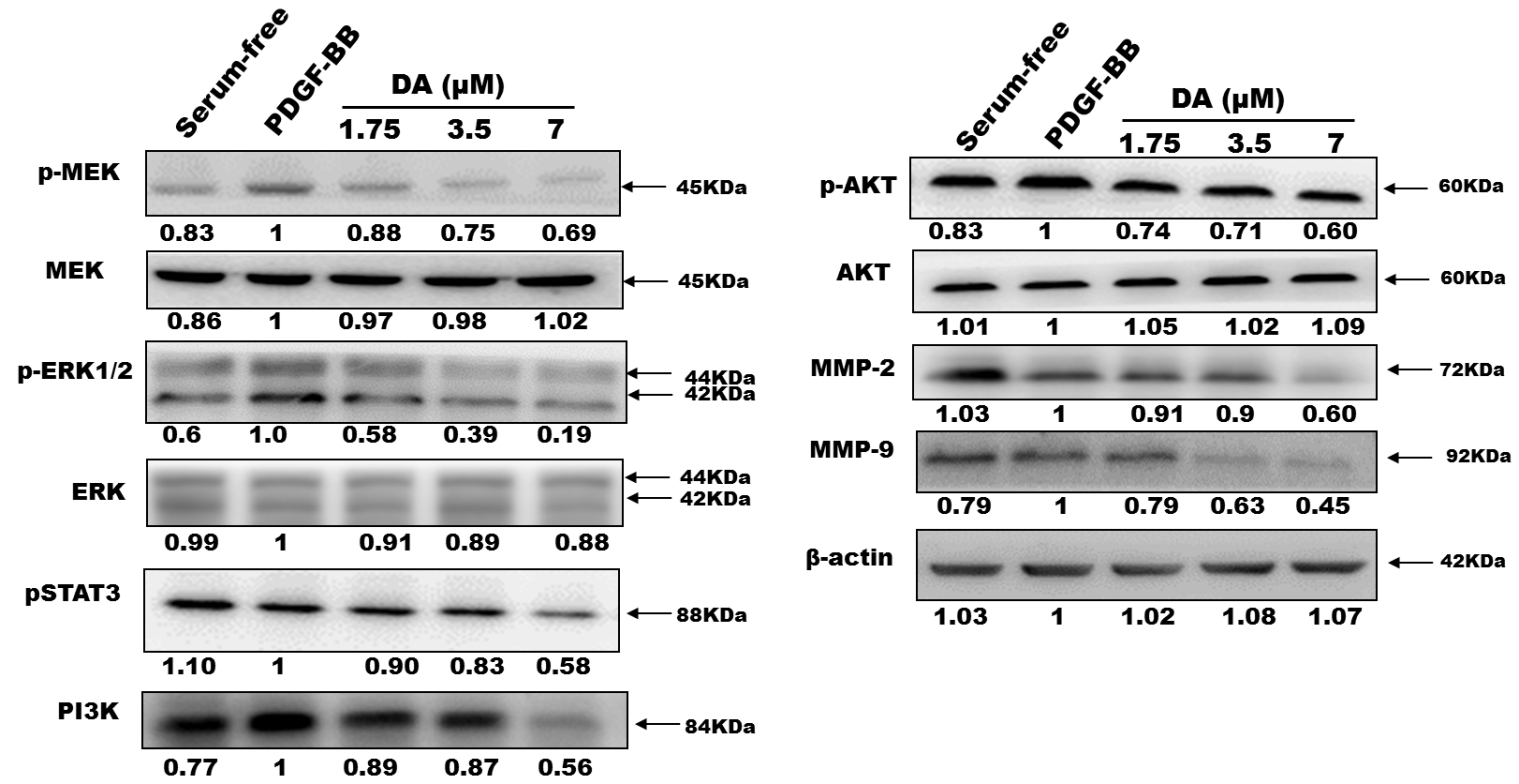
Dihydroaustrasulfone alcohol inhibited PDGF-BB-activated signaling cascades in VSMCs. VSMCs were treated with the indicated concentrations (1.75, 3.5 and 7 μM) in the presence of PDGF-BB (20 ng/mL). The expressions of detected proteins were normalized to the expression of the internal control (β-actin) and presented as relative expression to the control group (PDGF-BB alone).

We studied the underlying mechanisms of DA on PDGF-BB-induced VSMC migration and proliferation through Western blot analysis (Figure 4). After vascular injury, cell proliferation and migration of VSMCs were stimulated by released PDGF-BB, which plays an important role in vascular remodelling during cellular and extracellular responses to injury [15,16]. Several signaling pathways are involved in PDGF-BB-mediated cell proliferation and migration responses. Generally, ERK-MAPK and phosphoinositide 3-kinase (PI3K)/Akt cascades regulated cell proliferation and cell migration, respectively [17,18]. ERK1/2 is activated through the upstream molecule, mitogen-activated protein kinase (MEK). It has been found that ERK1/2 activation is rapidly upregulated after arterial injury and triggers a series of molecular events leading to neointima formation [19]. The inhibition of the ERK pathway has become a novel therapeutic strategy for reducing formation of neointimal hyperplasia [20]. In the present study, DA possessed significant effects in inhibiting activations of MEK and ERK1/2 in VSMCs stimulated with PDGF-BB (Figure 5).

Previous studies mentioned that PDGF-BB can induce cell migration *via* the PI3K/Akt and STATs pathways [21,22]. Activation of STAT3 by the Janus kinase-2 (JAK2) regulates gene expression in various biological processes, including cell proliferation, cell survival, and inflammation [23]. STAT3 is phosphorylated in response to growth factors and cytokines in a variety of proliferating cell types, including VSMCs [23,24]. In recent reports, STAT3 activation was linked to functional effects on neointimal cells, and inhibition of STAT3 signaling has been shown to antagonize these effects [21,24]. As shown in Figure 5, our data demonstrates that DA inhibited phosphorylation levels of both ERK and MEK proteins. Similarly, PDGF-BB-induced PI3K/AKT and STAT3 activations can also be significantly inhibited by DA (Figure 5).

Matrix metalloproteinases (MMPs), a family of Zn-dependent proteases that cleaves extracellular matrix structural proteins, may destabilize atherosclerotic plaques [25]. It has been reported that MMPs play key roles to regulate the migration activity of VSMCs. Expression and activation of MMP-2 constitutively expressed in VSMCs has been linked to VSMC migration and proliferation *in vitro* [26]. In addition, Cho *et al.* showed that MMP-9 also plays a critical role in migration of VSMCs and neointimal thickening. Their study found that MMP9 deficiency not only significantly reduced migration of VSMCs, but also down-regulated VSMC proliferation in MMP-9 null (MMP-9^−/−^) mice [27]. A previous report showed that gelatinases (MMP-2 and MMP-9) promoted neointima formation in animal models [28]. The results obtained from gelatin zymography assay suggested that DA dose-dependently decreased the pro-forms of all gelatinases as well as the active form of MMP9 as compared with the control group (PDGF-BB alone) (Figure 4). These results suggest that DA regulation of gelatinases may partially contribute to the inhibitory effects of DA on the migration and proliferation of VSMCs (Figure 5).

### 2.4. Dihydroaustrasulfone Alcohol Inhibited Neointimal Hyperplasia

**Figure 6:**
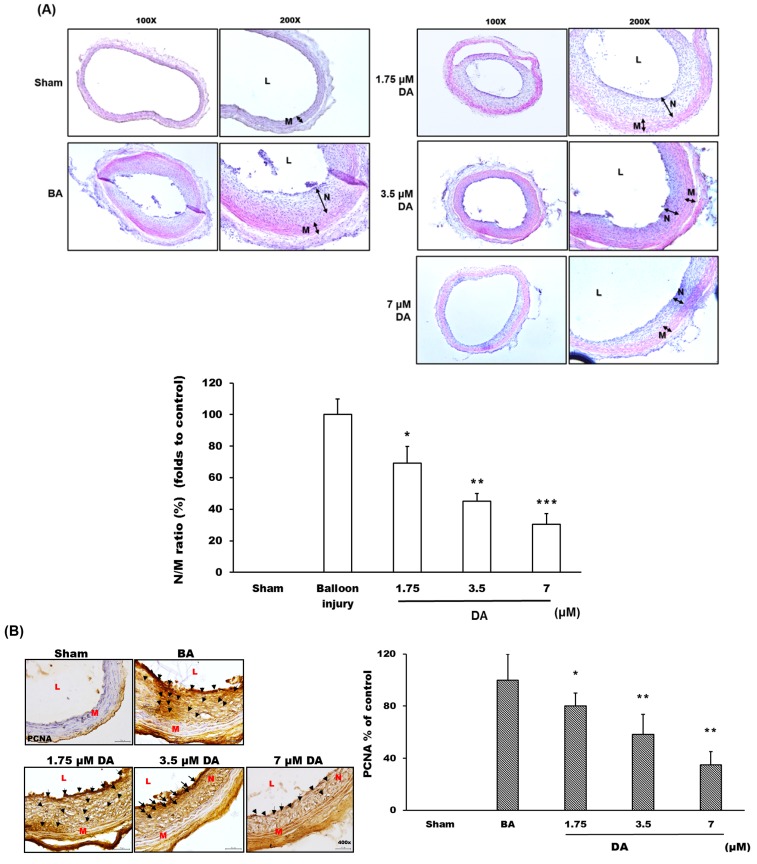

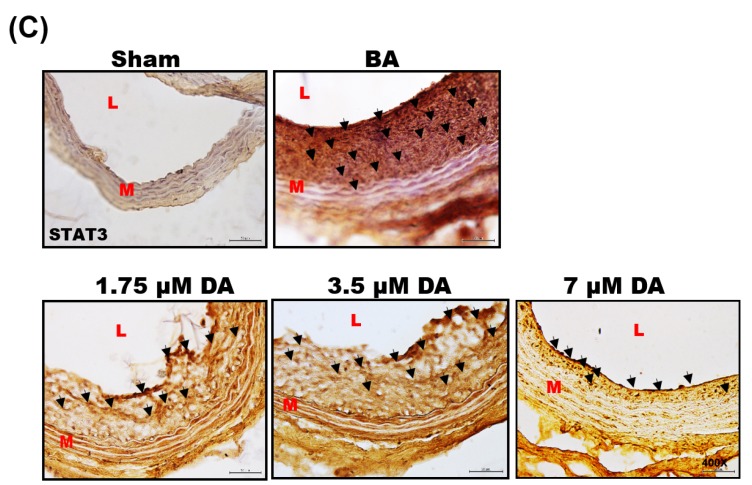
Dihydroaustrasulfone alcohol prevents balloon angioplasty-induced neointima formation. (**A**) The arterial sections were stained with hematoxylin-eosin (H&E) to observe the thickness changes of vessel wall. The images were acquired by microscopy at 100–200× magnification. The manifestation of vascular restenosis was presented as the ratio of neointima-to-media area (N/M ratio); (**B**) The distribution and expression of proliferating cell nuclear antigen (PCNA) protein were detected with immunohistochemistry analysis, and the images were acquired by microscopy at 400× magnification; (**C**) The distribution and expression of signal transducer and activator of transcription 3 (STAT3) protein were detected with immunohistochemistry analysis, and the images were acquired by microscopy at 400× magnification. L, lumen; N, neointima layer; M, media layer. The black arrow indicated the position with the expression of detected proteins.* *p* < 0.05, ** *p* < 0.01 and *** *p* < 0.001 compared with balloon angioplasty (BA) group, respectively.

After balloon angioplasty for two weeks, rat carotid arteries were collected and harvested to examine the histopathological changes in the arterial wall (Figure 6A). The balloon angioplasty procedure (BA group) significantly induced neointimal formation when compared with the sham group (Figure 6A). A statistical analysis results showed that the ratio of the neointima-to-media area (N/M ratio) of the BA group was increased compared with the sham group (Figure 6A). However, the groups treated with DA (1.75, 3.5 and 7 μM) (Figure 6A) exhibited a marked inhibition in neointima formation based on the change of N/M ratio (Figure 6A). These results suggested that DA treatment had an inhibitory effect on the progression of neointima formation in the rat carotid artery balloon injury model. Also, DA may inhibit and participate in the treatment of neointima formation (Figure 6A).

A previous study has demonstrated that the VSMC proliferation is a major character associated with vascular restenosis formation following balloon injury [10]. It is important to look for a simple, sensitive and specific evaluation index of cell proliferation. When resting cells begin to proliferate, the synthesis of proliferating cell nuclear antigen (PCNA) is activated and significantly increased, which is an important biological indicator of proliferation of responding cells [29]. PCNA has been investigated in clinical and basic studies of vascular restenosis following balloon injury [30]. Our results showed that PCNA expression was markedly increased in the neointima and media layers after balloon angioplasty, and DA treatment significantly reduced the expression of PCNA within the vessel wall (Figure 6B).

## 3. Experimental Section

### 3.1. Materials

DA was prepared by the reaction of 2-mercaptoethanol with methyl vinyl ketone to produce the corresponding sulfide, followed by oxidation of this sulfide with m-chloroperoxybenzoic acid (Figure 1) [9] PDGF-BB was obtained from Pepro Tech, Inc. (Rocky Hill, NJ, USA). RNase A, 3-(4,5-dimetylthiazol-2-yl)-2,5-diphenyltetrazolium bromide (MTT), propidium iodide (PI), trypsin, bovine serum albumin (BSA), Tween-20, Tween-80 and dimethyl sulfoxide (DMSO) were purchased from Amresco Inc. (Solon, OH, USA). Dulbecco’s modified Eagle’s medium (DMEM) and fetal bovine serum (FBS) were purchased from GIBCO BRL (Rockville, MD, USA). Cell culture supplies were purchased from Costar (Corning Inc., Cypress, CA, USA). The antibodies were purchased from Cell Signaling Technology, Inc. (Beverly, MA, USA) and Santa Cruz Biotechnology Co. (Santa Cruz, CA, USA).

### 3.2. Cell Culture and MTT Cell Proliferation Assay

VSMCs isolated from the thoracic aortas of 4–6 week-old Sprague-Dawley rats were maintained in DMEM medium supplemented with 10% FBS, 2 mM l-glutamine, 1 mM sodium pyruvate, 100 units/L penicillin, and 100 mg/L streptomycin. The cells were kept in a humidified 5% CO_2_–95% air incubator at 37 °C. The cells were used at passages 3–6 in the present study.

VSMCs seeded in 96-well plates (8 × 10^3^ cells/well) were treated with different concentrations of DA in the presence of PDGF-BB (20 ng/mL) for 24 h. After that, the culture medium was replaced with 100 μL of MTT (0.5 mg/mL) and then incubated at 37 °C. After 4 h incubation, the culture medium was aspirated and replaced with 100 μL of DMSO. The colorimetric intensity of formazan was quantified using an ELISA reader at 590 nm. The percentage of cell viability was calculated according to the values of the control group (PDGF-BB alone) as 100%.

### 3.3. Flow Cytometry

VSMCs seeded in 6-well plates (3 × 10^5^ cells/well) were treated with different concentrations of DA in the presence of PDGF-BB (20 ng/mL) for 24 h. After that, the trypsin-harvested cells were fixed in 70% ethanol, washed twice with PBS, and then incubated for 30 min with the staining solution containing 40 μM/mL of PI, 1% Triton-X 100 and 0.1 mg/mL RNase A. The fluorescence was measured and analysed using the FACScan flow cytometer (Becton Dickinson, San Jose, CA, USA).

### 3.4. Cell Migration Assay

For the wound healing assay, VSMCs were seeded in 6-well plates (3 × 10^5^ cells/well) and grown to confluence. After serum starvation, the cells were scraped to create a wound area in the centre of the cell monolayers (time point set as 0 h). Each well was washed once with PBS, and the cells were stimulated with 20 ng/mL of PDGF-BB and co-incubated with DA (1.75, 3.5 and 7 μM) for 18 h. After that, cells were washed once with PBS and fixed with 100% menthol for 15 min. The cells were stained with a Giemsa solution, and cells that migrated into the original wound area (at 0 h) were counted using Image J (Bethesda, MD, USA). The percentage of cell migration was calculated according to the values of the control group (PDGF-BB alone) as 100%.

For the transwell assay, 24-well plates mounted with ThinCert™ cell culture inserts (8.0-μm pore-size, Greiner Bio-one, Monroe, NC, USA) were used. Serum-free medium supplemented with 20 ng/mL of PDGF-BB was added in the plate wells and serum-free medium with various concentrations (1.75, 3.5 and 7 μM) of DA was added to the inside of the inserts seeded with VSMCs. After 18 h treatment, the migrated cells chemoattracted by PDGF-BB on the bottom (outside) of the inserts were stained with a Giemsa solution. The average number of migrated cells was counted from five randomly chosen regions of each insert using a microscope (OLYMPOS, Tokyo, Japan). The percentage of cell migration was calculated according to the values of the control group (PDGF-BB alone) as 100%.

### 3.5. In-Gel Gelatinase Zymography

The culture medium was harvested to examine gelatinase activity using 10% SDS-PAGE gel electrophoresis with 0.2% gelatine under non-reducing conditions. After electrophoresis, the gelatinases were renatured by rinsing the gel in 2.5% Triton X-100 at room temperature (RT) for 30 min and then incubated in the reaction buffer (2 M Tris-HCl, pH 8.0, 1 M CaCl_2_ and 1% NaN_3_) at 37 °C for 18 h. The gels were stained with 0.25% Coomassie brilliant blue R250 for 90 min and then destained with 10% acetic acid in 40% methanol. The activated gelatinases were visualized as clear bands on the blue-stained gels. The clear bands were quantified by using the Multi Gauge v3.0 software. The percentages of gelatinase activities were calculated according to the values of the control group (PDGF-BB alone) as 100%.

### 3.6. Western Blot

The harvested cells were lysed with PRO-PREP^®^ protein extraction solution (iNtRON Biotechnology, Sungnam, South Korea). Protein extracts (30 μg of total protein/sample) were electrophoresed using 10% SDS-PAGE and then transferred to the polyvinylidene difluoride (PVDF) membranes (BioTrace™, Ann Arbor, MI, USA). The blotted membranes were blocked in 5% non-fat milk for 1 h and then incubated overnight at 4 °C with the primary antibodies against MEK (#sc6250; Santa Cruz Biotechnology, Santa Cruz, CA, USA), phospho-MEK (#9121; Cell Signalling Technology, Beverly, MA, USA), PI3K (#ab63040; Abcam Ltd., Cambridge, UK), AKT (#sc-5298; Santa Cruz Biotechnology), phospho-AKT (#4060; Cell Signalling Technology), ERK1/2 (#sc292838; Santa Cruz Biotechnology), phospho-ERK1/2 (#sc7383; Santa Cruz Biotechnology), phospho-STAT3 (#GTX61820; GeneTex, Irvine, CA, USA), MMP-2 (#ab37150; Abcam Ltd.), and MMP-9 (#ab19016; Millipore, Billerica, MA, USA). After that, the membranes were further incubated with the horseradish peroxidase-linked secondary antibodies (Gene Tex, USA) for 1 h, followed by signal visualization using an electrochemical luminescence (ECL) reagent. Images were acquired using the Image Quant LAS4000 gel imager (Fujifilm Life Science, Tokyo, Japan). Band intensities were quantified by using the Multi Gauge v3.0 software (Fujifilm Life Science, Tokyo, Japan). The percentage of protein expression was calculated according to the values of the control group (PDGF-BB alone) as 100%.

### 3.7. Rat Carotid Artery Balloon Angioplasty

Male Sprague Dawley (SD) rats (approximately 250~300 g) were purchased from BioLASCO Taiwan Co. Ltd. (Taipei, Taiwan) and housed with a 12-h light/dark cycle with free access to food and water. All experimental procedures involving animals were approved by the ethics committee of the Institutional Animal Care and Use Committee (IACUC) of China Medical University (Permit Number: 101-146N, Date: 10 May 2013). All animal care followed the institutional animal ethical guidelines of China Medical University. Surgery was performed under Zoletil^®^ anesthesia (Milperra, NSW, Australia). The rats were randomly divided into five groups (*n* = 6/group): Sham control, Balloon angioplasty (BA), and BA + DA (1.75, 3.5 or 7 μM). Angioplasty of the rat carotid artery was performed as described previously [10]. The balloon catheter (2F Fogarty; Becton-Dickinson, Franklin Lakes, NJ, USA) was introduced through the left external carotid artery into the common carotid artery, and the balloon was inflated at 1.3 kg/cm^2^ using an inflation device. The inflated balloon was pushed and pulled through the lumen three times to damage the arterial wall. DA was dissolved in 30% (*w*/*v*) of pluronic gel F-127, and then well-mixed gels were coated around the outside of the arterial segment injured by balloon angioplasty. After 14 days, the rats were sacrificed with an overdose of Zoletil^®^ anesthesia, and bilateral common carotid arteries were harvested for further pathological examinations.

### 3.8. Histopathological Analysis

The harvested carotid artery was fixed in 4% paraformaldehyde solution for 24 h, embedded in paraffin, and cut into 7-mm transverse sections. After that, the tissue sections were either subjected to hematoxylin-eosin (H&E) staining or immunohistochemical staining. After routine H&E staining, the morphological changes of the vessel wall in each section were analysed with computer-based analyser software (Image J) to calculate the ratio of the neointima-to-media area (N/M ratio). The neointimal layer was defined as the region between the vessel lumen and the internal elastic fibres within the vascular wall, and the media layer was defined as the region between the internal and external elastic fibres.

Immunohistochemical analysis was carried out using the DAKO system (#K0679; Dako LSAB + System-HRP, DAKO, Tokyo, Japan). First the tissue sections were rehydrated and immersed in 3% hydrogen peroxide for 30 min to quench the endogenous peroxidase, and then all sections were further incubated in 1% BSA for 1 h at RT. Subsequently, the sections were incubated with the primary antibodies against PCNA (#sc56; Santa Cruz Biotechnology, USA) and phospho-STAT3 overnight at 4 °C. After that, the sections were incubated with the biotinylated antibody and peroxidase-labelled streptavidin for 30 min at RT. For signal detection, the sections were incubated with the ready-to-use DAB substrate-chromogen solution for 5 min according to the manufacturer’s protocol. Lastly, the sections were counterstained with haematoxylin and mounted with hard-set media (Assistant-Histokitt, Sondheim, Germany). Photomicrographs were obtained using a microscope (OLYMPOS, Japan) at 200× and 400× magnification.

### 3.9. Statistical Analysis

All data are presented as the mean ± standard error of mean (SEM) and analysed by using the SPSS v18.0 statistical package (SPSS, Chicago, IL, USA). One-way ANOVA was carried out to evaluate statistical difference among multiple groups. A value of *p* < 0.05 was considered statistically significant.

## 4. Conclusions

**Figure 7:**
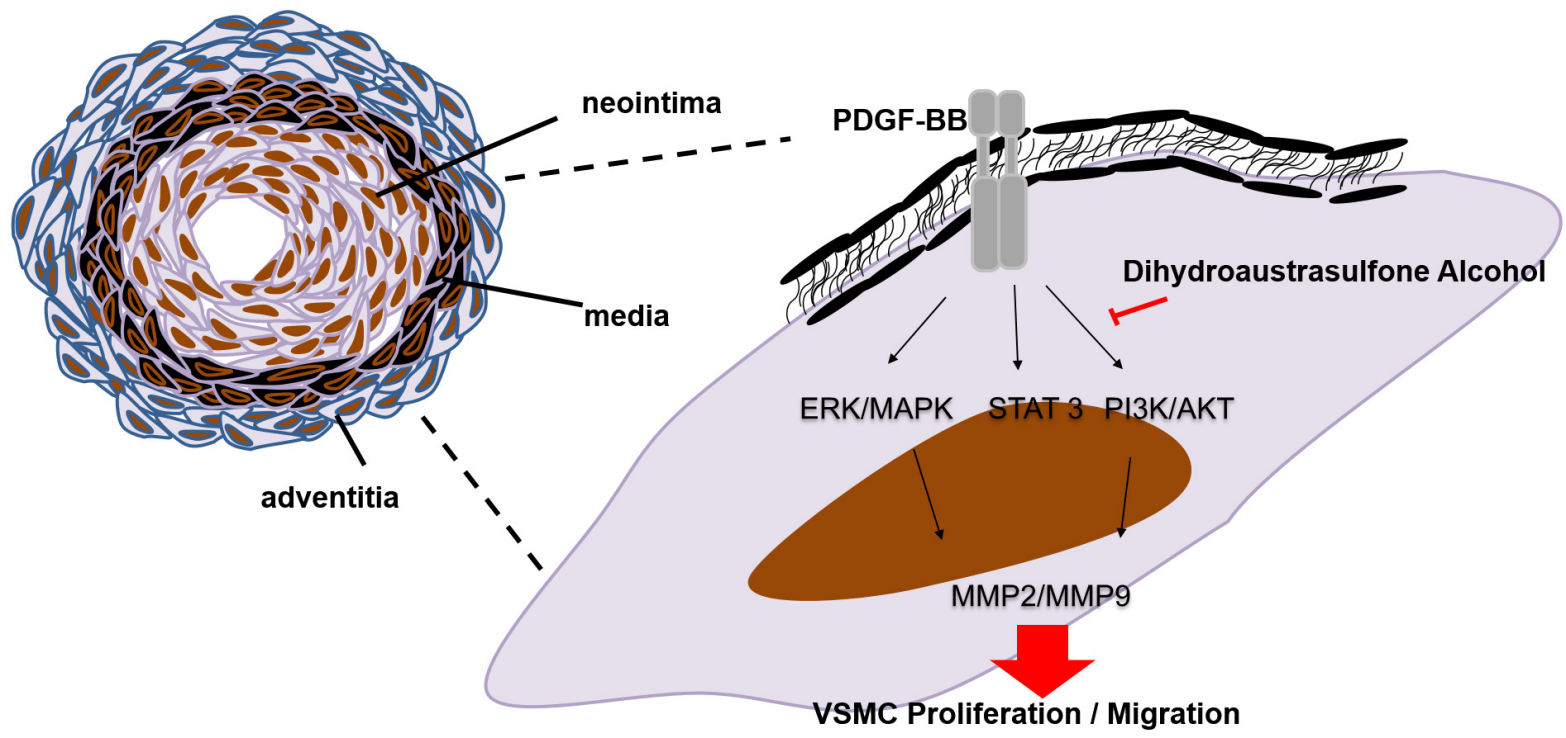
Schematic representation of the potential mechanisms of Dihydroaustrasulfone Alcohol in regulating PDGF-BB-induced VSMC proliferation and migration. PDGF-BB, platelet-derived growth factor-BB; ERK/MAPK, extracellular signal-regulated kinase/mitogen-activated protein kinase; PI3K/AKT, phosphoinositide-3-kinase/protein kinase B; STAT3, signal transducer and activator of transcription 3; MMP2/MMP9, matrix metalloproteinases 2/9; VSMC, vascular smooth muscle cell. Red arrow: regulation signaling pathways. Black arrow: inhibit signaling pathway.

This study provides the first evidence that DA can inhibit PDGF-BB-induced VSMC migration and proliferation, the effect of which may inhibit the activations of MEK/ERK and PI3K/AKT cascades and STAT3 as well as PCNA expression (Figure 5, Figure 6B and Figure 7). The results obtained from *in vivo* study indicate that inhibitory effects of DA in PDGF-activated cellular responses may provide partial explanations for why DA can prevent the development of balloon injury-induced neointimal hyperplasia. Based on these data, it may be a good therapeutic strategy to prevent balloon injury-induced restenosis by targeting the PDGF-BB-mediated pathway. These results suggest that DA has a potential application as an anti-restenotic agent for the prevention of restenosis.
